# Prevalence profile perceived stress and impact on quality of life for recurrent aphthous ulcers: a cross-sectional epidemiological study in Egypt

**DOI:** 10.1186/s12903-026-08151-7

**Published:** 2026-04-15

**Authors:** Dalia Ghalwash

**Affiliations:** Oral Medicine and Periodontology, Faculty of Dentistry, The British University in Egypt, El Sherouk City, Cairo, 11837 Egypt

**Keywords:** Recurrent aphthous ulcer, Prevalence, Stress, Impact, Quality of life

## Abstract

**Background:**

Recurrent Aphthous Ulcerations (RAU) are among the most frequent oral ulcers with unknown etiology and multiple risk factors. This study assessed the prevalence profile of aphthous ulcers, risk factors, levels of perceived stress, and their impact on quality of life in an Egyptian sample.

**Methods:**

This study involved screening 4100 individuals from the outpatient clinic, Faculty of Dentistry, The British University in Egypt, and several Egyptian governorates. Patients were subjected to clinical examination and patient interviews for accurate diagnosis and collection of data. The Perceived Stress Scale (PSS) was used to assess levels of perceived stress, and the Oral Health Impact Profile-5 (OHIP-5) was used to assess oral health-related quality of life.

**Results:**

RAU affected 2.6% of the studied sample, 62.9% were females, and 37.1% were males, 33.3% of cases fell in the age range of 15 to 24, and 44.8% were 25 to 34 years. The minor type represented 83.8% of RAU cases, the major type represented 11.4%, and the herpetiform type represented 4.8%. High perceived stress was reported in 49.5% of cases, and moderate levels at 45.7%. PSS score was significantly correlated with RAU’s frequency, duration, and pain intensity, and the OHIP-5 score was significantly correlated with RAU’s duration and pain intensity.

**Conclusion:**

The level of perceived stress substantially influences the frequency, duration, and pain intensity of RAU episodes, which are correlated with a worse effect on the patient’s quality of life; thus, stress management would be beneficial in reducing the negative effect of RAU on the quality of the patient’s life.

**Supplementary Information:**

The online version contains supplementary material available at 10.1186/s12903-026-08151-7.

## Introduction

 Recurrent Aphthous Ulcerations (RAU) are the most frequent inflammatory oral mucosal ulcers. It affects around 20% of the general population, causes discomfort, and affects daily activities [1]. Ulcers are painful, self-limited, of unknown etiology, with several underlying risk factors, recurring 3 to 6 times annually. They are more common in females and high socioeconomic levels [2, 3].

There are three types of aphthous ulcers: major, minor, and herpetiform RAU. The minor form is the most frequent, accountable for up to 85% of all RAU cases, they are shallow, circular, or ovoid ulcers, have yellow floors, are covered with a grayish-yellow pseudo-membrane, and are bounded by erythematous haloes, with a diameter of less than 1 cm and heal within 10–14 days [4]. The major form represents 10% to 15%, with a diameter of more than 1 cm, usually single, deeper, indurated, and can last for months, leaving scars and causing significant pain and dysphagia [4]. The herpetiform RAU rarely occurs and appears as numerous pinpoint ulcers (5-100) that may coalesce, forming larger, irregular lesions lasting 7 to 14 days [4, 5].

The etiology and pathogenesis of RAU remain unknown. Still, it primarily involves activating T lymphocytes, which produce excessive amounts of cytokines, inducing the death of epithelial cells and tissue destruction [3, 6]. Hereditary factors, hematological aberrations, infections, trauma, gastrointestinal diseases, hormonal factors, nutritional deficiencies, and psychological stress may all be involved in its progression [5, 7].

Since the definitive pathogenesis of RAU is still vague, a definitive cure is not available. The main goals of therapy are pain relief, reduction of duration of lesions, accelerating the healing process, with decreasing the frequency of RAU episodes. Common treatment options for RAU patients include topical or systemic corticosteroids, immunosuppressives, antibiotics, anti-inflammatory drugs, and analgesics [2, 5].

Aphthous ulcers on nonkeratinized oral mucosa commonly impact the lips, tongue, buccal mucosa, and soft palate. They can cause substantial pain and may make eating, speaking, and swallowing difficult, which can, therefore, affect the quality of life [3].

Recognizing the prevalence of RAU in the general population is necessary because it helps understand the percentage affected by the disease and the associated risk factors in a specific population, as the ulcers have significant negative effects on oral health and can affect the quality of life. Epidemiological studies have found significant variances in the prevalence of RAU (2% to 66%) in different populations [7]. Epidemiological studies are rare in Egypt, however, an earlier Egyptian study reported an RAU prevalence rate of 1.15% [8].

Even though RAU is a frequent condition, only a few studies have investigated the impact of RAU on quality of life [9, 10]. Accordingly, this study aimed to assess the prevalence of different variants of aphthous ulcers in an Egyptian sample, possible relation to risk factors, the level of perceived stress, and their impact on quality of life.

## Materials and methods

### Study design

The present study is a cross-sectional observational epidemiological study, coducted during the period from November 2023 to November 2024, Research Ethics Committee of Faculty of Dentistry, the British University in Egypt approved the study protocol with approval No. (REC 24–043). All patients, or their parents if younger than 18, signed consent forms.

#### Sample size

The EPI INFO program were initially used, but due to the low prevalence of the condition under study, the resulting sample size was small. Therefore, the present study included all available cases that met the inclusion criteria during the study period, which extended over one year from November 2023 to November 2024, resulting in a total of 4100 subjects. It is a convenience sample of all eligible individuals encountered during the study period.

#### Sampling criteria

Inclusion Criteria: Egyptians, both males and females, aged 15 years and older were included.

Exclusion Criteria: Non-Egyptians and patients under 15 years of age were excluded.

#### Recruitment

Subjects were recruited consecutively from the outpatient clinic of the Faculty of Dentistry, The British University in Egypt, and also from rural and civilized locations in Cairo Governorate, Al-Fayoum Governorate, Al-Sharkia Governorate, and Gharbia Governorate, as part of convoys funded and supported by The British University in Egypt. Consecutive sampling was undertaken to minimize selection bias.

After initial screening, patients complaining of oral aphthous ulcers were subjected to a detailed clinical examination to diagnose RAU [4], and patient interviews included specific questions, a questionnaire was developed for this study and uploaded as supplementary file-1, regarding age, sex, frequency of appearance, the usual duration, location, and pattern of the ulcer.

Questionnaire content validity was assessed by a gory of 3 experts in the field of dermatology. Questions of the studied questionnaire were assessed for relevance and clarity, with the item validity index being excellent for all questions, ranging from 0.92 to 1. The scale validity index was assessed to be 0.95.

CVI cut-off.

If I-CVI:

> 0.79, it is considered appropriate.

0.7–0.79 needs revision.

< 0.7, it is deleted.

If S-CVI: was more than or equal to 0.90, the scale was judged as a valid tool.

Finally, pain severity was assessed by the Visual Analogue Scale (VAS) score. Risk factors include family history, hormonal factors, trauma, history of systemic diseases (hypertension, diabetes mellitus, gastrointestinal disorders, anemia) were reported, and smoking habit where smokers were classified into 3 categories: light smokers (smoking 1–10 cigarettes/day), moderate smokers (smoking 11–20 cigarettes/day), and heavy smokers (smoking 20 cigarettes/day). Additionally, the perceived stress was assessed using the Perceived Stress Scale (PSS), which is one of the most widely used stress assessment methods, especially the shortened 10-item version (PPS-10),

The questions are: l. In the last month, how often have you been upset because of something that happened unexpectedly? 2. In the last month, how often have you felt that you were unable to control the important things in your life? 3. In the last month, how often have you felt nervous and stressed? 4. In the last month, how often have you felt confident about your ability to handle your problems? 5. In the last month, how often have you felt that things were going your way? 6. In the last month, how often have you found that you could not cope with all the things that you had to do? 7. In the last month, how often have you been able to control irritations in your life? 8. In the last month, how often have you felt that you were on top of things? 9. In the last month, how often have you been angered because of things that happened that were outside of your control? 10. In the last month, how often have you felt difficulties were piling up so high that you could not overcome them? Scores can range from 0 to 40, from 0 to 13 indicates low stress, from 14 to 26 indicates moderate stress, and from 27 to 40 indicates high perceived stress [11–13].

The impact on quality of life was evaluated using the Oral Health Impact Profile-5 (OHIP-5), which only included five questions: (1) Have you had any difficulty chewing food because of problems with teeth, mouth, denture, or jaw? (2) Have you had any painful aching in your mouth? (3) Have you felt uncomfortable about the appearance of your teeth, mouth, denture, or jaw? (4) Have you felt that there has been less flavor in your food because of problems with your teeth, mouth, denture, or jaw? (5) Have you had difficulty performing your everyday activities because of problems with your teeth, mouth, denture, or jaw? And responses were made as five categories (1 = never, 2 = hardly ever, 3 = occasionally, 4 = fairly often, and 5 = very often). OHIP-5 scores range from 0 to 20, with higher scores indicating worse perceived oral health status and impact on quality of life [14]. An Arabic version of OHIP-5 has been validated [15].

### Statistical analysis and data interpretation

SPSS software, version 26 (SPSS Inc., PASW statistics for Windows version 26. Chicago: SPSS Inc.) was used for data analysis. Qualitative data were defined using numbers and percentages. Quantitative data were described using median (minimum and maximum) for non-normally distributed numerical data and mean±standard deviation for normally distributed data after testing normality using the Kolmogorov-Smirnov test. The significance of the results obtained was adjusted at the 0.05 level. The chi-squared test was used to compare categorical data between groups as appropriate. The Mann-Whitney U test was used to compare the 2 studied groups for non-normally distributed numerical data. Student t-test was used to compare 2 independent groups for normally distributed numerical data. One-way ANOVA test was used to compare numerical data from more than 2 independent groups, with the Post Hoc Tukey test to detect pair-wise comparison. Spearman’s rank-order correlation determines the strength and direction of a linear relationship between two non-normally distributed numerical datasets.

## Results

**Fig. 1:**
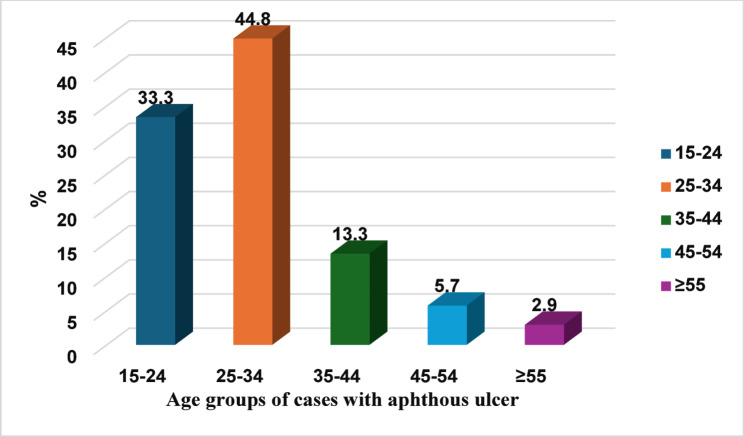
Distribution of RAU cases according to age groups

The current cross-sectional study screened 4100 subjects, aged 15 to 60 years, with a mean of 30.1 ± 8.98, 59.7% males and 40.3% females. The diagnosis of aphthous ulcers was made in 105 cases representing a prevalence of 2.6% of the sample studied, 35 cases fell in the age range of 15 to 24, and 47 cases were 25 to 34 years old, while only 14 cases were between 35 and 44 years, 6 cases from 45 to 54, and 3 cases only were older than 55 (Fig. 1). Thirty-nine RAU cases were males (37.1%) and 66 were females (62.9%).

**Fig. 2:**
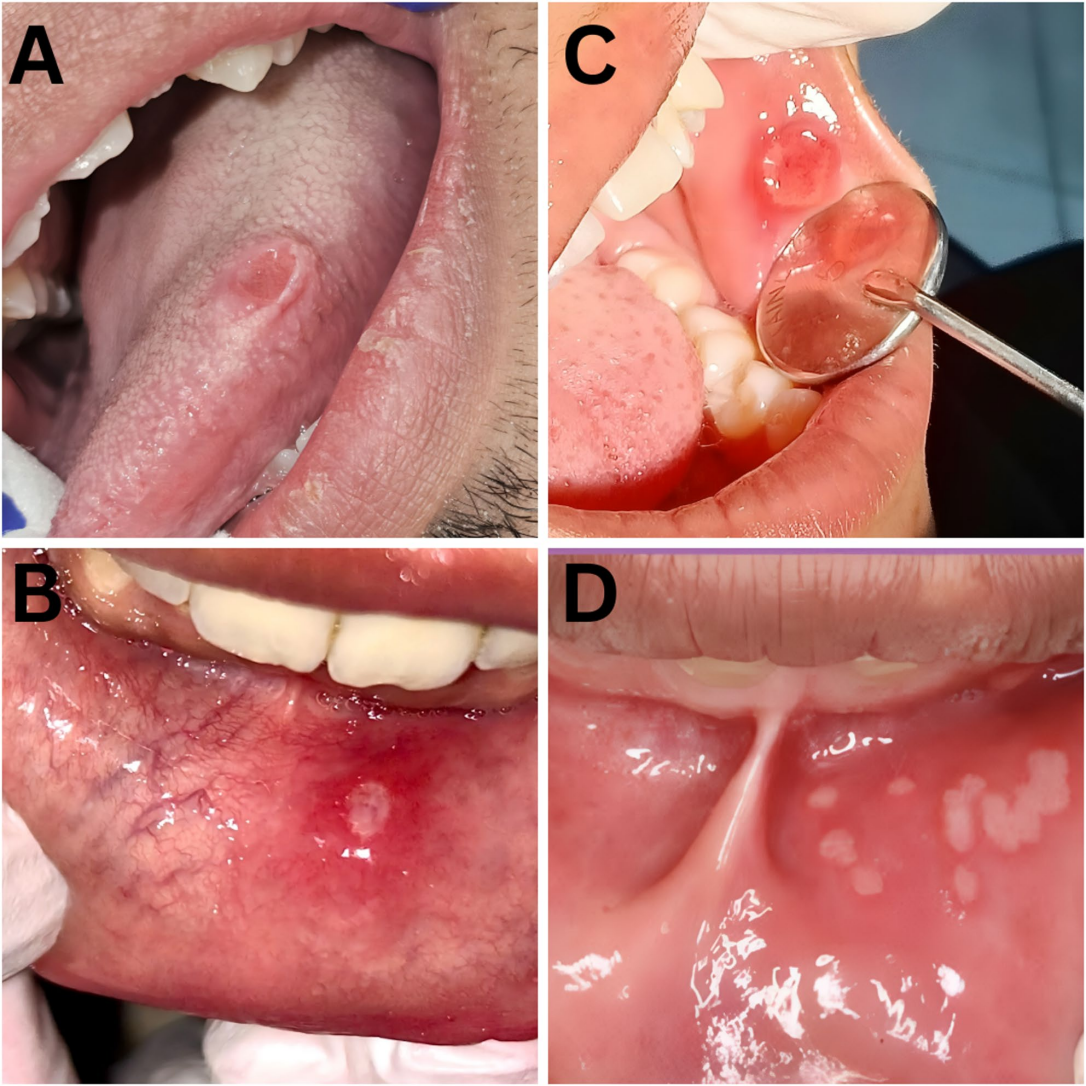
**A**. 21-year-old male with a minor aphthous ulcer on the lateral border of the tongue. (**B**) 19-year-old female with a minor aphthous ulcer on the labial mucosa. (**C**) 25-year-old female with major aphthous ulcer on the buccal mucosa. (**D**) 22-year-old female with herpetiform aphthous ulcer in the labial mucosa and vestibule

**Fig. 3:**
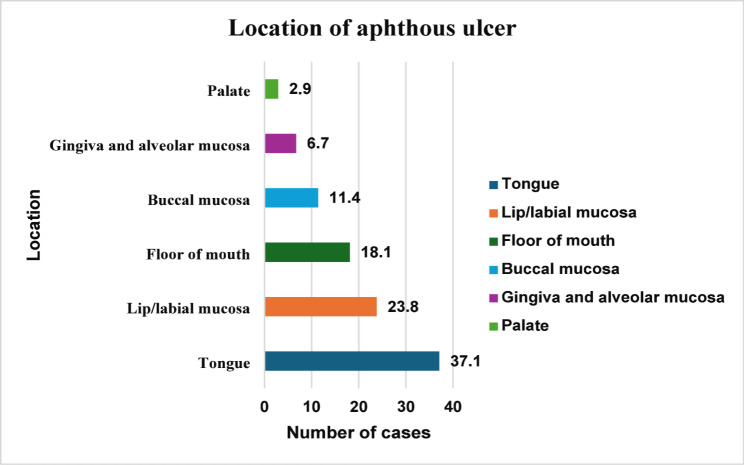
Distribution of RAU cases according to locations

Table 1 demonstrates all the descriptive data of RAU cases. Minor aphthous ulcers were the most common type encountered in 88 patients, representing 83.8% of RAU cases (Fig. 2). On the other hand, major aphthous ulcers were found in 12 patients, representing 11.4% of RAU cases, and the herpetiform type was only encountered in 5 patients, representing 4.8% of RAU cases. The most commonly involved site was the tongue, involved in 37.1% of RAU cases, followed by the lip and labial mucosa, involved in 23.8% of cases, the floor of the mouth, involved in 18.1%, and the buccal mucosa in 11.4%. In contrast, the gingiva and alveolar mucosa were only affected in 6.7% of cases, and the palate was affected in 2.9% of RAU cases (Fig. 3). The most commonly reported frequencies for RAU were three, five, and four times per year, reported by 35.2%, 24.8%, and 20% of RAU cases, respectively. The reported duration of RAU was less than 7 days in 19% of patients, between 7 and 14 days in 57.1% of cases, and more than 14 days in 23.8% of cases. A history of systemic diseases was found in 41 cases, and 91.4% of cases were nonsmokers. A positive family history of RAU was reported in 69 cases (65.7%), and hormonal factors were reported in 16.2% of cases.

**Table 1:** Descriptive data of RAU cases

	*N = 105*	%
Smoking frequency		
Nonsmoker	96	91.4
Light	4	3.8
Moderate	2	1.9
Heavy	3	2.9
Clinical Type		
Minor Major RAU Herpetiform RAU	88 12 5	83.8 11.4 4.8
Location		
Tongue Palate Lip/labial mucosa Gingiva and alveolar mucosa Floor of mouth Buccal mucosa	39 3 25 7 19 12	37.1 2.9 23.8 6.7 18.1 11.4
Frequency/year	3(1–6)	
1 2 3 4 5 6	2 15 37 21 26 4	1.9 14.3 35.2 20.0 24.8 3.8
Duration (days)		
< 7 7–14 > 14	20 60 25	19.0 57.1 23.8
VAS		
Mean ± SD (Range)	5.27 ± 1.18 (4–9)	
Systemic diseases	*N = 41*	
Hypertension	9	22.0
Gastrointestinal disorders	13	31.7
Diabetes mellitus	8	19.5
Anemia	11	26.8
Family history	69	65.7
Hormonal factors	17	16.2

Results of the PSS score of RAU cases revealed that high levels of perceived stress were reported in 52 cases (49.5%), and moderate levels of perceived stress in 48 cases (45.7%). On the other hand, low levels of perceived stress were reported in only 5 cases (4.8%). The relation between demographic and clinical characteristics with perceived stress among cases with aphthous ulcers was displayed in Table 2, showing a significant association between high levels of perceived stress and the major and herpetiform types, frequency, and duration of RAU, as well as the OHIP-5 score.

**Table 2:** Relation between demographic and clinical characteristics with perceived stress level among RAU cases

	Stress		Test of significance
	Low to moderate *N* = 53	High *N* = 52	
Age/ years	29.68 ± 9.87	29.98 ± 9.98	t = 0.361 *p* = 0.719
Gender			
Male Female	22(41.5) 31(58.5)	17(32.7) 35(67.3)	ꭓ^2^=0.874 *p* = 0.350
Smoking frequency			
Nonsmoker Light Moderate Heavy	47(88.7) 3(5.7) 1(1.9) 2(3.8)	49(94.2) 1(1.9) 1(1.9) 1(1.9)	ꭓ^2^=1.36 *p* = 0.714
Clinical Type			
Minor Major RAU Herpetiform RAU	52(98.1) 0 1(1.9)	36(69.2) 12(23.1) 4(7.7)	ꭓ^2^=16.70 *p* = 0.001*
Location			
Tongue Palate Lip/labial mucosa Gingiva and alveolar mucosa Floor of mouth Buccal mucosa	15(28.3) 1(1.9) 12(22.6) 6(11.3) 12(22.6) 7(13.2)	24(46.2) 2(3.8) 13(25) 1(1.9) 7(13.5) 5(9.6)	ꭓ^2^=7.66 *p* = 0.176
Frequency/year	3(1–5) 3.11 ± 0.97	4(2–6) 4.15 ± 1.12	Z = 4.48 *p* = 0.001*
Duration (days)			
< 7 7–14 > 14	18(34) 29(54.7) 6(11.3)	2(3.8) 31(59.6) 19(36.5)	ꭓ^2^=19.62 *p* = 0.001*
VAS	5(4–6) 4.96 ± 0.62	5(4–9) 5.58 ± 1.5	Z = 1.52 *p* = 0.129
OHIP-5	4(3–6) 4.23 ± 0.69	5(3–8) 5.21 ± 1.45	Z = 3.59 *p* = 0.001*
Systemic diseases			
Hypertension	0	9(34.6)	ꭓ^2^=7.48 *p* = 0.058
Gastrointestinal disorders	7(46.7)	6(23.1)	
Diabetes mellitus	4(26.7)	4(15.4)	
Anemia	4(26.7)	7(26.9)	
Family history			
Negative	22(41.5)	14(26.9)	ꭓ^2^=2.48
Positive	31(58.5)	38(73.1)	*p* = 0.115
Hormonal factors			
Negative	47(88.7)	41(78.8)	ꭓ^2^=1.87
Positive	6(11.3)	11(21.2)	*p* = 0.171

*t* Student t-test, ꭓ ^2^Chi-Square test, *statistically significant Z: Mann-Whitney U test

Table 3 demonstrates the relation between demographic and clinical characteristics and the quality-of-life score among RAU cases, where a significant association was encountered with the major followed by the herpetiform types of RAU, and the location on the palate.

**Table 3:** Relation between demographic and clinical characteristics with quality-of-life score among RAU cases

	OHIP-5 Mean ± SD	Test of significance
Gender		
Male Female	4.51 ± 1.14 4.83 ± 1.27	t = 1.29 *p* = 0.199
Smoking frequency		
Nonsmoker Light Moderate Heavy	4.69 ± 1.25 4.75 ± 0.96 6.0 ± 1.41 4.67 ± 0.58	F = 0.740 *p* = 0.530
Clinical Type		
Minor Major RAU Herpetiform RAU	4.31 ± 0.72^AB^ 7.5 ± 0.522^AC^ 5.20 ± 0.45^BC^	F = 114.48 *p* = 0.001*
Location		
Tongue Palate Lip/labial mucosa Gingiva and alveolar mucosa Floor of mouth Buccal mucosa	4.54 ± 1.16^A^ 6.67 ± 1.52^ABCDE^ 4.72 ± 1.1^B^ 4.14 ± 1.1^C^ 4.74 ± 1.37^D^ 5.08 ± 1.24^E^	F = 2.32 *p* = 0.048*
Systemic diseases		
Hypertension Gastrointestinal disorders Diabetes mellitus Anemia	4.44 ± 0.52 5.31 ± 1.70 5.63 ± 1.51 5.27 ± 1.68	F = 1.03 *p* = 0.391
Family history		
Negative Positive	4.53 ± 1.21 4.81 ± 1.24	t = 1.12 *p* = 0.264
Hormonal factors		
Negative Positive	4.63 ± 1.09 5.18 ± 1.74	t = 1.71 *p* = 0.091

*t* Student t-test, F One Way ANOVA test, *statistically significant

Similar superscripted letters in the same column denote significant differences between studied subcategories

**Fig. 4:**
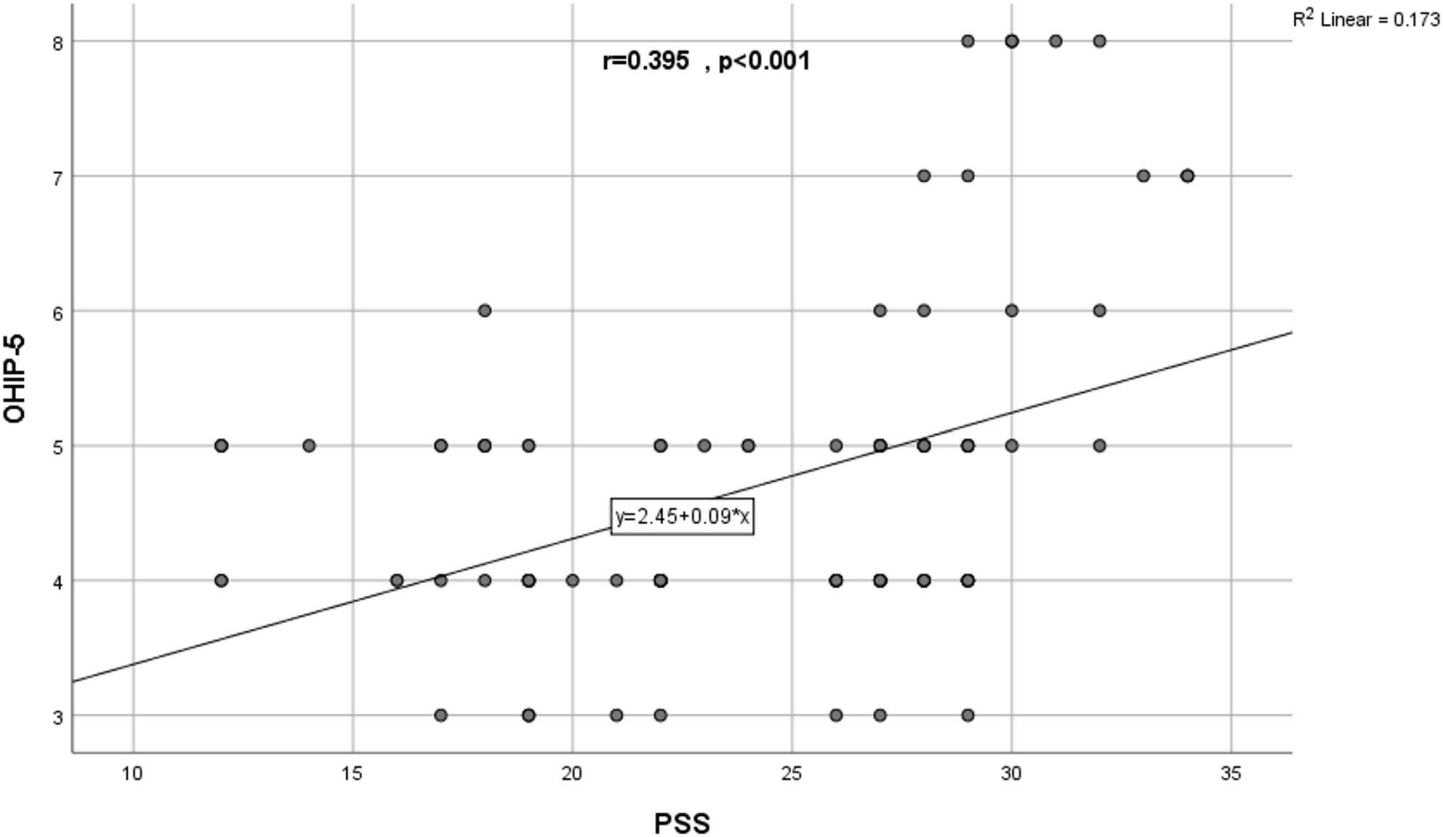
A scatter diagram showing the correlation between PSS & OHIP-5 among the studied RAU cases

A significant correlation was found between the PSS score and the frequency and duration of RAU and VAS scores. At the same time, the OHIP-5 score was significantly correlated with the duration of the RAU and VAS scores as presented in Table 4. Figure 4 illustrates a scatter diagram showing the correlation between PSS & OHIP-5 among the studied RAU cases.

**Table 4:** Correlation between OHIP-5 and PSS score with age, frequency, duration, and VAS score among RAU cases

	OHIP-5		PSS score	
	*r*	*p*	*r*	*p*
Age/ years	0.037	0.707	-0.005	0.958
Frequency/year	0.076	0.444	0.507	0.001*
Duration (days)	0.331	0.001*	0.559	0.001*
VAS scores	0.687	0.001*	0.245	0.012*

## Discussion

Identifying the frequency of RAU is essential because it helps understand the percentage of individuals affected by the disease and the associated risk factors in a specific population. Various epidemiological studies have found significant variances in the prevalence of RAU (20% to 66%) in different populations [7, 16]. Different study methodologies, socioeconomic levels, environmental factors, populations, and sample sizes significantly influence RAU’s prevalence.

In the current study, 105 cases were diagnosed having RAU out of 4100 subjects, examined over a whole year at the Faculty of Dentistry outpatient clinic at The British University in Egypt in addition to several rural and civilized locations in Egypt to increase the generalizability of the study, representing a prevalence of 2.6% of the studied sample. The diagnosis of RAU was made after patient interviews and a detailed oral examination. A previous study at Cairo University found that RAU was prevalent at 1.15%, using patient interviews and comprehensive clinical examinations, just like the present investigation [8]. A retrospective study of RAU cases revealed a prevalence of 1.755% [5]. Additionally, a study conducted in India recorded an overall RAU prevalence of 0.78% [17].

In some studies, the prevalence rate was higher; India had a prevalence of 21.7% [18]. Similarly, the prevalence of 28.2% was estimated in Iraq [19]. A recent study revealed that 47.2% of college students were affected by RAU [7]. The higher prevalence reported in those studies could be related to using questionnaires in which RAU could be mistaken for other lesions. In addition, patients reported that they have experienced RAU in the past and are not currently affected by RAU, as in the present study. To accurately measure the prevalence of RAU, it is important to combine clinical examination with questionnaires in future studies.

The prime age of RAU occurrence is mainly in the young to middle age, as 33.3% of patients in the current study fell in the age range of 15 to 24, and 44.8% of cases were 25 to 34 years. This agrees well with several studies [10, 17, 19]. Females made up 62.9% of RAU cases in this study, which agrees with many studies that have reported higher prevalence in females [10, 17–19]. The greater prevalence of women has been linked to hormonal factors according to some authors. In contrast, limited investigations have described a higher male frequency [20].

The majority of RAU cases occurred in nonsmokers (91.4%), which is consistent with previous research that RAU occurs less frequently in smokers [19, 21]. Smoking has the potential to protect the oral mucosa by enhancing keratinization, which acts as a mechanical and chemical barrier against trauma or microbes [3, 21]. Additionally, nicotine stimulates the secretion of adrenal steroids by its action on the hypothalamic adrenal axis, reducing the production of tumor necrosis factor ∝ (TNF-∝), interleukin-1 (IL-1), and interleukin-6 (IL-6), thus inhibiting the inflammatory response and tissue destruction [9]. The development of RAU may be influenced by psychological stress, which may be lower in smokers [8, 22].

Minor aphthous ulcers were the most common type encountered in 83.8% of RAU cases. In contrast, 11.4% of RAU cases had major aphthous ulcers, and 4.8% had the herpetiform type, which is consistent with most literature [4, 7, 9, 16, 20, 21]. The most commonly involved site was the tongue, involved in 37.1% of RAU cases, followed by lip and labial mucosa, in line with another study, the tongue was the most affected region, followed by the buccal mucosa, while the floor of the mouth and palate were not as affected [5]. According to other studies, ulceration was most common in the lips and buccal mucosa [7, 19].

Gastrointestinal disorders were the most common condition among patients in the current study, followed by anemia, hypertension, and diabetes. Various studies agree that systemic conditions are a risk factor in many RAU cases [19, 22–24]. A positive family history of RAU was reported in 69 cases (65.7%), which is consistent with previous research [24]. This might be reinforced by the reported genetic links between RAU and human leukocyte antigens (HLA) [24, 25]. Hormonal factors were reported in 16.2% of cases in the present research, which is supported by some studies reporting a correlation between menstruation and aphthous ulcers [26]; however, other studies state that this claim is not sufficiently supported [27].

Psychological stress has been suggested by many studies as a possible cause of RAU’s onset and progression. Across the present results, 49.5% of cases had high levels of perceived stress, while 45.7% had moderate levels of stress. Additionally, high levels of perceived stress were significantly associated with the major and herpetiform types of aphthous ulcers, which are more painful and disturbing to patients, as well as a high OHIP-5 score indicating the negative influence of stress on the patient’s quality of life.

The PSS score had a significant correlation with the frequency, duration of RAU, and pain intensity. A recent study suggests that over half of RAU cases experience moderate-to-high stress levels, and previous studies have shown the significant burden of mental health on RAU patients [28, 29]. Psychological stress was considered a significant initiating factor of RAU [30]. In contrast, some studies found no correlation between stress and RAU episodes [31, 32].

The present results agree with earlier studies reporting that pain intensity is associated with the level of psychological stress in RAU cases [33–35], and that a bigger number of major RAU patients recorded moderate to high stress compared to minor RAU which could be because the major type is deeper, larger, lasts longer, more painful, and causes substantial functional impairment, leading to more psychological impact than the minor type. However, they found no impact of increased stress levels on the frequency or duration of the RAU episodes [16, 33]. In contrast, a previous study conveyed that stress influenced the duration of RAU [36], and other studies reported that higher stress levels are associated with an increase in RAU episodes [5, 7], which is in line with the present findings.

The underlying mechanism linking stress with RAU is not well understood. The concentration of salivary cortisol increases during stress, leading to the recruitment of leukocytes to inflammatory sites, which is crucial for the development of RAU. Genetic changes in immune pathways related to stressful responses could be implicated, which include the activation, distribution, and proliferation of lymphocytes and natural killer cells, as well as cytokines and antibody production [4]. Such immune system alterations have also been linked to RAU, and this may partially explain the role of stress in the development of RAU [10].

Oral diseases significantly impact the oral health-related quality of life [37–39]. To assess oral health-related quality of life, the present study utilized OHIP-5, which is an informative and low-burden alternative to the most popular OHIP-14 [40]. In the present study, the major type of RAU, being larger, deeper, and lasting longer than other types followed by the herpetiform type, and the location of the ulcer on the palate had the worst impact on the patient’s quality of life, which could be because the palate is densely packed with sensory nerve endings making it highly sensitive to irritation, also the location of RAU on the palate makes it more difficult to avoid during normal oral movements and can cause significant discomfort, especially when talking, eating, or drinking.

Moreover, the OHIP-5 score was significantly correlated with the duration of the RAU and VAS scores. Indicating that the increase in pain intensity and duration of RAU episodes had the most adverse effect on quality of life, which is in line with earlier research concluding that RAU increased the negative impacts of oral health on patients’ quality of life [9, 13, 35]. Similarly, another study stated that higher levels of stress and anxiety were associated with worse oral health impacts of ulcers and inferior quality of life [41, 42], which is in line with the present results.

It is noteworthy that high stress levels and poor quality of life were associated with a negative history of trauma, which means that in cases of traumatic ulcers, stress is usually not an underlying cause, and the patient’s quality of life would not be affected as much as it is affected by RAU.

In light of this association between the patient’s perceived stress levels and the oral symptoms of RAU and the impact of the condition on the patient’s quality of life, the management of RAU should include psychological assessment and referring the patients for appropriate management which could help improve the disease activity and quality of life of RAU as stress may trigger the mechanisms involved in the pathogenesis of RAU, leading to pain and negative effects on daily functions, initiating a cycle in which stress leads to ulcers and ulcers lead to stress [41]. Also, the association between pain intensity and quality of life highlights the importance of relieving pain in treating RAU.

The present study’s strengths lie in its large sample size and the inclusion of cases from multiple governorates, which makes it more representative and transferable to the real-world population, enhancing the generalizability of the results. Both patient interviews and clinical examinations were implemented to improve the validity and reliability. The limitations included the probability of information bias concerning risk factors and systemic diseases during data collection and the difficulty in performing randomization, so consecutive sampling was undertaken to decrease selection bias. An additional limitation is that correlations can only be identified in cross-sectional studies, but not causal relations, whether stress causes RAU or is a consequence of having it, socioeconomic factors were not assessed presently, so it might be a potential confounder.

## Conclusion

The present results revealed an RAU prevalence of 2.6%. The majority of cases were females in the 15 to 34-year-old age range. High levels of perceived stress were reported in 49.5% of cases and moderate levels in 45.7%, indicating a significant burden of stress among patients with RAU, which makes screening for perceived stress a sensible practice in those patients. The level of perceived stress was found to substantially influence the frequency, duration, and pain intensity of RAU, which are associated with a worse impact on the quality of life; thus, stress management interventions would be beneficial in reducing the negative effect of RAU on the quality of life.

## Supplementary Information

Supplementary Material 1.

## Data Availability

The data is available from the corresponding author upon reasonable request.
