# Supplementary material for: Prevalence profile perceived stress and impact on quality of life for recurrent aphthous ulcers: a cross-sectional epidemiological study in Egypt
Source: BMC Oral Health. 2026 Apr 15;26:781. doi: 10.1186/s12903-026-08151-7 (PMC13135698; doi:10.1186/s12903-026-08151-7)
Supplement: Supplementary file 1 — Supplementary Material 1. [file 12903_2026_8151_MOESM1_ESM.docx]

| 1. **Do you agree to participate in this study?** | 1. Yes 2. No |
| --- | --- |
| 1. **Name** |  |
| 1. **Age** |  |
| 1. **Sex** | 1. Male 2. Female |
| 1. **Smoking frequency:** | 1. No 2. Light smokers (1-10 cigarettes/day) 3. Moderate smokers (11-20 cigarettes/day) 4. Heavy smokers (≥20 cigarettes/day) |
| 1. **Systemic condition** | 1. Hypertension 2. Diabetes Mellitus 3. Cardiovascular disease 4. Anemia 5. Gastrointestinal disorder. 6. Others 7. No |
| 1. **Does any of your family members have a history of similar lesions appearing in the oral cavity (RAU)?** | 1. Yes 2. No |
| 1. **Do you associate the appearance of these lesions with any hormonal factors such as menstruation or pregnancy?** | 1. Yes 2. No |
| 1. **Do you associate the appearance of these lesions with occurrence of trauma to oral mucosa?** | 1. Yes 2. No |
| 1. **The usual location of RAU** | 1. Tongue 2. Lip/labial mucosa 3. Buccal mucosa 4. Gingiva and alveolar mucosa 5. Floor of the mouth 6. Palate |
| 1. **Frequency of RAU appearance** | 1. 1 time per year 2. 2 times per year 3. 3 times per year 4. 4 times per year 5. 5 times per year 6. 6 times per year 7. >6 times per year |
| 1. **The usual duration of RAU** | 1. <7 days 2. 7-14 days 3. >14 days |
| 1. **The usual clinical type of RAU** | 1. Minor 2. Major 3. Herpetiform |
| 1. **Intensity of pain (VAS)**   No pain  **0 1 2 3 4 5 6 7 8 9 10** | The worst pain imaginable. |
| 1. **OHIP-5 score:** |  |

1. **Perceived stress questionnaire total score:**
